# Introducing FDG PET/CT-guided chemoradiotherapy for stage III NSCLC in low- and middle-income countries: preliminary results from the IAEA PERTAIN trial

**DOI:** 10.1007/s00259-019-04421-5

**Published:** 2019-07-31

**Authors:** T. Konert, W. V. Vogel, D. Paez, A. Polo, E. Fidarova, H. Carvalho, P. S. Duarte, A. C. Zuliani, A. O. Santos, D. Altuhhova, L. Karusoo, R. Kapoor, A. Sood, J. Khader, A. Al-Ibraheem, Y. Numair, S. Abubaker, C. Soydal, T. Kütük, T. A. Le, N. X. Canh, B. Q. Bieu, L. N. Ha, J. S. A. Belderbos, M. P. MacManus, D. Thorwarth, G. G. Hanna

**Affiliations:** 1 Nuclear Medicine Department, Netherlands Cancer Institute, Plesmanlaan 121, 1066 CX Amsterdam, The Netherlands; 2 Department of Radiation Oncology, Netherlands Cancer Institute, Amsterdam, The Netherlands; 3 Division of Human Health, Department of Nuclear Sciences and Applications, International Atomic Energy Agency, Vienna, Austria; 4 Department of Radiology and Oncology, Faculty of Medicine, University of São Paulo – Institute of Cancer of Sao Paulo State, São Paulo, Brazil; 5 Department of Radiation Oncology and Nuclear Medicine Department, Hospital das Clínicas, Campinas University, Campinas, Brazil; 6 Department of Radiation Oncology and Radiology Department, North Estonia Medical Center, Tallinn, Estonia; 7 Department of Radiation Oncology and Nuclear Medicine Department, Postgraduate Institute of Medical Education and Research, Chandigarh, India; 8 Department of Radiation Oncology and Nuclear Medicine Department, King Hussein Cancer Center, Amman, Jordan; 9 Department of Radiation Oncology and Nuclear Medicine Department, Institute of Nuclear Medicine and Oncology, Lahore, Pakistan; 10 Department of Radiation Oncology and Nuclear Medicine Department, Ankara University School of Medicine, Mamak/Ankara, Turkey; 11 Department of Radiation Oncology and Nuclear Medicine Department, Cho Ray Hospital, University of Ho Chi Minh City, Ho Chi Minh City, Vietnam; 12 Department of Radiation Oncology and Radiosurgery, Tran Hung Dao Hospital, Hanoi, Vietnam; 13 Department of Radiation Oncology, Peter MacCallum Cancer Centre, 305 Grattan Street, Melbourne, VIC 3000 Australia; 14 Sir Peter MacCallum Department of Oncology, University of Melbourne, Parkville, Australia; 15 Section for Biomedical Physics, Department of Radiation Oncology, University Hospital Tübingen, Tübingen, Germany

**Keywords:** Low- and middle-income countries, Non-small-cell lung cancer, PET/CT-guided chemoradiotherapy

## Abstract

**Purpose:**

Patients with stage III non-small-cell lung cancer (NSCLC) treated with chemoradiotherapy (CRT) in low- and middle-income countries (LMIC) continue to have a poor prognosis. It is known that FDG PET/CT improves staging, treatment selection and target volume delineation (TVD), and although its use has grown rapidly, it is still not widely available in LMIC. CRT is often used as sequential treatment, but is known to be more effective when given concurrently. The aim of the PERTAIN study was to assess the impact of introducing FDG PET/CT-guided concurrent CRT, supported by training and quality control (QC), on the overall survival (OS) and progression-free survival (PFS) of patients with stage III NSCLC.

**Methods:**

The study included patients with stage III NSCLC from nine medical centres in seven countries. A retrospective cohort was managed according to local practices between January 2010 and July 2014, which involved only optional diagnostic FDG PET/CT for staging (not for TVD), followed by sequential or concurrent CRT. A prospective cohort between August 2015 and October 2018 was treated according to the study protocol including FDG PET/CT in treatment position for staging and multimodal TVD followed by concurrent CRT by specialists trained in protocol-specific TVD and with TVD QC. Kaplan–Meier analysis was used to assess OS and PFS in the retrospective and prospective cohorts.

**Results:**

Guidelines for FDG PET/CT image acquisition and TVD were developed and published. All specialists involved in the PERTAIN study received training between June 2014 and May 2016. The PET/CT scanners used received EARL accreditation. In November 2018 a planned interim analysis was performed including 230 patients in the retrospective cohort with a median follow-up of 14 months and 128 patients in the prospective cohort, of whom 69 had a follow-up of at least 1 year. Using the Kaplan–Meier method, OS was significantly longer in the prospective cohort than in the retrospective cohort (23 vs. 14 months, *p* = 0.012). In addition, median PFS was significantly longer in the prospective cohort than in the retrospective cohort (17 vs. 11 months, *p* = 0.012).

**Conclusion:**

In the PERTAIN study, the preliminary results indicate that introducing FDG PET/CT-guided concurrent CRT for patients with stage III NSCLC in LMIC resulted in a significant improvement in OS and PFS. The final study results based on complete data are expected in 2020.

**Electronic supplementary material:**

The online version of this article (10.1007/s00259-019-04421-5) contains supplementary material, which is available to authorized users.

## Introduction

Worldwide, lung cancer is the most commonly diagnosed cancer (2.1 million new cases in 2018) and the leading cause of cancer death (1.8 million deaths estimated in 2018) [1]. Five-year survival of lung cancer was 20–33% in countries such as Japan, Canada, USA, China, Korea, Israel, Sweden, Switzerland and Austria. However, most other countries had a 5-year survival ranging between 10% and 20%. Survival was less than 10% in countries such as Brazil, India and Thailand. Globally, lung cancer survival rates between 1995 and 1999 and between 2000 and 2014 indicate no improvement with time, but in high-income countries 5-year overall survival (OS) has increased by 5–10% in absolute terms over the same time period [2].

Currently, ^18^F-fluorodeoxyglucose positron emission tomography/computed tomography (FDG PET/CT) is widely used for staging patients with non-small-cell lung cancer (NSCLC) and to a lesser extent for radiotherapy (RT) target volume delineation (TVD) [3, 4]. PET/CT scanners have also become available in several low- and middle-income countries (LMIC), although FDG PET/CT is mainly used for staging purposes rather than as a part of treatment planning in NSCLC [5]. The International Atomic Energy Agency (IAEA) convened an expert panel to appraise the clinical utility of FDG PET/CT for staging and RT planning (RTP) in patients with lung cancer. This coordinated research programme resulted in the design of the international PET/CT in RTP (PERTAIN) study (NCT02247713) to assess the feasibility of including FDG PET/CT in the RTP process in patients with stage III NSCLC in LMIC.

The current standard treatment for stage III NSCLC is concurrent chemoradiotherapy (CRT) [6]. In order to take advantage of the recent developments in RT techniques which have improved the accuracy of treatment delivery, it is essential to ensure TVD is as accurate as possible to avoid geographic miss of disease. Advanced RT techniques have improved local tumour control and have reduced treatment toxicity by enabling the delivery of higher radiation doses to the tumour while sparing adjacent normal tissue [7]. Examples include intensity-modulated RT (IMRT) [8], and image-guided RT, which improves the precision of treatment delivery and allows the use of smaller expansion margins [9].

TVD involves contouring the gross tumour volume (GTV), as specified in ICRU report 50 [10]. GTV delineation is sensitive to interobserver variability (IOV) [11, 12]. A significant reduction in IOV can be achieved with information from both PET and CT [12–15]. Automatic PET segmentation methods have also been proposed to reduce IOV [16], but always need verification by a radiation oncologist (RO) [17]. PET is specifically helpful in TVD when the tumour is not easily distinguished from surrounding healthy tissue on CT images, due to its higher soft tissue contrast [18]. Even with the use of PET imaging there is still IOV due to differences in the TVD method and to FDG uptake in normal structures adjacent to the tumour [19, 20]. The use of a rigorous contouring protocol in which a multidisciplinary team including a RO and a nuclear medicine physician (NMP) follow a detailed set of instructions has been shown to help minimize IOV [21]. A recent IAEA publication has provided guidance on the use and role of FDG PET/CT imaging for RTP in NSCLC patients [17]. The impact of the use of the IAEA study protocol on TVD accuracy and reproducibility has been evaluated in multiple centres in LMIC. Multiple training interventions on PET/CT-based TVD in NSCLC improves delineation accuracy and reduces IOV [16]. Hence, we hypothesized that TVD following the IAEA study protocol would increase accuracy and reproducibility of TVD in the clinic, leading to improvement in local control.

There are many reports describing IOV within and outside the context of clinical trials, but few studies have investigated the impact of IOV on clinical outcome [20, 22, 23] or methods that could minimize IOV by means of training [16, 24–26]. The clinical impact of such training remains unknown. Hence, we hypothesized that TVD following the IAEA study protocol would increase the accuracy and reproducibility of TVD and lead to improvement in local control and thus OS. We present the preliminary results of the PERTAIN study. The aim of this study was to assess the impact of introducing FDG PET/CT-guided concurrent CRT, supported by training and quality control (QC), on the OS and PFS in patients with stage III NSCLC.

## Materials and methods

### Ethical aspects

The PERTAIN study was approved by the medical ethics committee of Netherlands Cancer Institute/Antoni van Leeuwenhoek Hospital (ref. M14PRI). In addition, each centre received ethical clearance from their local medical research ethics committee. Written informed consent was obtained from all patients included in the prospective phase.

### Study framework

Nine medical centres in seven countries met the technical requirements to participate in the PERTAIN study, including six middle-income countries: Brazil, India, Jordan, Pakistan, Turkey and Vietnam. The seventh country, Estonia, is classified as a high-income country by the World Bank, but did not routinely use PET/CT for RTP. The first component of the study was data collection from a retrospective cohort, which included consecutive patients with stage III NSCLC who had been treated in the participating centres between January 2010 and July 2014 according to existing local protocols. Over a 1-year period, nine pairs of trainees each including a RO and a NMP with limited experience in PET/CT-based TVD in NSCLC from seven different countries took part in multiple training interventions. Teams were given hands-on training in delineating the primary tumour according to IAEA protocol guidelines. An online webinar training session was held on TVD in NSCLC, and lectures for ROs and NMPs on current best practice in NSCLC were given [19]. All PET/CT scanners received annual European Association of Nuclear Medicine (EANM) Research Ltd. (EARL) FDG PET/CT accreditation. After the training intervention and scanner calibration, patients with stage III NSCLC were included in the prospective cohort between August 2015 and October 2018. The study entry criteria are summarized in the Supplementary material S1 (Form 1). Patients who did not meet the study entry criteria were excluded from the study.

### Case report forms

Patient data were collected using electronic case report forms (eCRFs). Five different eCRFs were designed to collect information on patient eligibility, before and after treatment, and follow-up. More details on eCRFs and their format can be found in the Supplementary material S1.

### Clinical endpoints

The primary endpoint was OS, defined as the time between the start of treatment and date of death or loss to follow-up. The secondary endpoint was progression-free survival (PFS). PFS was defined as the time from the start of treatment to local failure, time to regional failure, and/or time to distant failure. Local failure was defined as progression in the primary tumour, and regional failure as progression in involved lymph nodes as assessed on follow-up scans. Distant failure was defined according to the 8th edition of the TNM classification for NSCLC [27]. The intervals for the follow-up assessments and imaging were as per local follow-up guidelines.

### Chemotherapy and radiotherapy details

Patients in the retrospective cohort were treated according to respective institutional practice with sequential concurrent CRT, neoadjuvant chemotherapy or RT alone, but with curative intent. In the prospective cohort, patients were treated with concurrent CRT to a total dose of at least 60 Gy in fractions of 2 Gy over 6 weeks. Centres were free to select chemotherapy regimens according to local practice.

### PET/CT image acquisition

Patients underwent whole-body FDG PET/CT using one of the following scanners: Discovery ST, Discovery 710, Discovery STE (GE Medical Systems, Chicago, IL, USA), Biograph 40 mCT, and Biograph 64 mCT (Siemens Medical Solutions, Erlangen, Germany). The reconstruction voxel size of the PET data varied from 2.0 × 2.0 × 3.3 mm to 5.5 × 5.5 × 3.3 mm. Patients fasted for at least 8 h to ensure low levels of serum glucose. The total injected dose ranged between 226 MBq and 441 MBq (data not available for all patients). Patients were scanned approximately 60 min after injection of ^18^F-FDG according to EANM guidelines [28]. The acquisition times of the PET/CT scanners were in the range 2–5 min per bed position.

### Assessment of the retrospective and prospective cohort

To assess the overall impact of the multiple training interventions and the routine use of FDG PET/CT-based concurrent CRT, survival outcomes in the retrospective cohort were compared with those in the prospective cohort. Although the training programme focused mainly on standardized PET/CT-based TVD, in general, the whole RTP procedure was also standardized to ensure the use of current treatment standards. Differences in the RTP procedures between the retrospective and prospective cohorts are summarized in Table 1.

**Table 1:** Differences in staging, radiotherapy planning, treatment and target volume delineation procedures between the retrospective and prospective cohorts

Comparison	Retrospective cohort	Prospective cohort
Staging	With or without PET/CT	With PET/CT
RTP	With or without PET/CT	PET/CT in RTP-position
Time interval	Per local protocol, delays of >1 month possible	Within 4 weeks of last PET/CT
Delivered dose	Per local protocol	≥60 Gy
Treatment	RT, sequential CRT, CCRT	CCRT only
TVD	Per local protocol	Per IAEA study protocol (PET/CT-based)
PET/CT quality assurance	EARL accreditation not compulsory	EARL accreditation compulsory
Nodal irradiation	Both elective and involved nodal RT	Involved nodal RT

All procedures in the prospective cohort were standardized in all centres in accordance with the IAEA study guidelines [17]

*(C)CRT*  concurrent) chemoradiotherapy, *RT* radiotherapy, *RTP* radiotherapy planning, *TVD* tumour volume delineation

### Quality control of target volume delineation

To ensure that participating centres in the prospective study complied with the IAEA study protocol, central QC review of TVD was performed for the first three patients included per centre, and thereafter as needed. In the QC process anonymized PET/CT data and RT structure sets were made available through a secure online storage service and were reviewed by at least two members of the study trial management group.

### Statistical analysis

Any differences in continuous variables between the retrospective and prospective cohorts were assessed using the independent *t* test. Any differences in categorical variables were assessed using the chi-squared test. Strong prognostic factors were identified using univariate Cox regression analysis. Kaplan–Meier analysis was performed to assess OS and PFS in the retrospective and prospective groups. The log-rank statistic was used to assess the significance of any differences. Statistical analysis was performed using IBM SPSS statistics for Windows, version 22.0 (IBM Corp., Armonk, NY). Values of *p* less than 0.05 were considered significant.

## Results

### Patient inclusion

The retrospective cohort included 230 patients with stage III NSCLC treated with sequential or concurrent CRT or RT alone. The prospective cohort included 69 patients with stage III NSCLC. In all centres, a high percentage of patients (up to 51%) were upstaged to stage IV after staging with PET/CT became the standard. Overall, five patients did not meet the study inclusion criteria, and were therefore excluded. Reasons for exclusion were inability to provide informed consent (one patient), unable to start treatment within 4 weeks of PET/CT (two patients), and an ECOG performance status (PS) of 2 (two patients). An overview of the patient and tumour characteristics is given in Table 2.

**Table 2:** Patient and tumour characteristics

	Retrospective cohort	Prospective cohort	*p* value^a^
No. of patients	230	69	–
Mean age (range)	61 (31–86)	64 (43–86)	0.136
Gender			
Male	191 (83%)	57 (83%)	0.831
Female	39 (17%)	12 (17%)	
Smoker	182 (79%)	67 (97%)	<0.001
COPD	75 (33%)	47 (68%)	<0.001
ECOG performance status			
0	72 (31%)	21 (30%)	0.841
1	158 (69%)	48 (70%)	
Disease stage			
IIIA	145 (63%)	29 (42%)	0.021
IIIB	53 (23%)	27 (39%)	
IIIC	32 (14%)	13 (19%)	
T stage			
1	4 (2%)	3 (4%)	0.369
2	43 (19%)	11 (16%)	
3	87 (38%)	21 (30%)	
4	96 (42%)	34 (49%)	
N stage			
0	16 (7%)	4 (6%)	0.082
1	39 (17%)	4 (6%)	
2	135 (59%)	42 (61%)	
3	40 (17%)	19 (27%)	
Histology			
Squamous cell carcinoma	90 (39%)	32 (46%)	0.013
Adenocarcinoma	97 (42%)	35 (51%)	
Large cell carcinoma	15 (7%)	0 (0%)	
Not otherwise specified	28 (12%)	2 (3%)	

*COPD* chronic obstructive pulmonary disease, *ECOG* Eastern Cooperative Oncology Group

^a^Calculated using the independent *t* test for continuous variables or the chi-squared test for categorical variables

### Quality control of target volume delineation

All participating centres completed the first step of the QC procedure, in which the first three patients were accepted in the PERTAIN study. In total, 35 patients were submitted for TVD QC. Nine patients (26%) were excluded after evaluation. The reasons for not accepting the TVD as acceptable were: incorrect staging (two patients), involved lymph nodes not included (two patients), tumours too large to treat radically (≥60 Gy) without exceeding dose constraints (three patients), and noncompliance with IAEA study guidelines (two patients). All other patients in the prospective cohort were accepted for inclusion and treatment with concurrent CRT.

### Treatment parameters

In the retrospective cohort, 18 patients were treated with RT only (8%), 65 patients received sequential CRT (28%), and 147 patients (64%) received concurrent CRT. In contrast, all patients in the prospective cohort received concurrent CRT with curative intent. In both the retrospective and prospective cohorts various chemotherapy regimens were intravenously administered weekly: either carboplatin-based or cisplatin-based in combination with paclitaxel, etoposide, docetaxel, pemetrexed or gemcitabine. Of the 230 patients in the retrospective cohort, 32 (14%) were treated using an IMRT technique and 198 (86%) using three-dimensional conformal RT (3DCT). By comparison, of the 69 patients in the prospective cohort, 29 (42%) were treated with IMRT, 2 (3%) with volumetric modulated arc therapy (VMAT), and 38 (55%) with 3DCT. The prescribed dose fractionation scheme varied between 50 Gy in 30 fractions and 70 Gy in 35 fractions in the retrospective cohort, with a mean prescribed dose of 61.4 ± 2.8 Gy. In the prospective cohort the dose fractionation scheme varied between 60 Gy in 30 fractions and 66 Gy in 33 fractions, with a mean prescribed dose of 60.7 ± 1.7 Gy.

### Impact on survival

Prognostic factors were evaluated in the retrospective and prospective cohorts separately. In the retrospective cohort, age and ECOG PS were significant prognostic factors (*p* = 0.039 and 0.024, respectively), and T stage demonstrated borderline significance (*p* = 0.053). In the prospective cohort, univariate Cox regression analysis showed no significant prognostic factors. No significant differences between the retrospective and the prospective cohorts in any of these prognostic variables were found, and therefore these variables were considered balanced. However, TNM staging was significantly higher in the prospective cohort than in the retrospective cohort (*p* = 0.021), and histological subtype was significantly different between the cohorts (*p* = 0.013) The difference in histological subtype was due to the absence of large-cell and lack of not otherwise specified types in the prospective cohort (see Table 2).

**Fig. 1:**
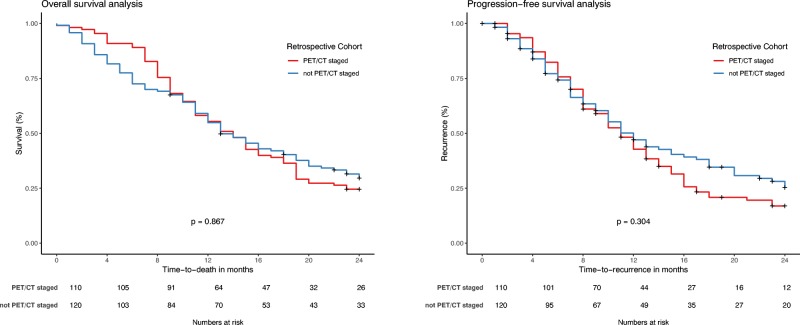
Kaplan–Meier analysis of the difference in overall survival (*left*) and progression-free survival (*right*) in the retrospective cohort between patients who were and were not PET/CT-staged. No significant differences were observed in overall survival (*p* = 0.867) or progression-free survival (*p* = 0.304)

**Fig. 2:**
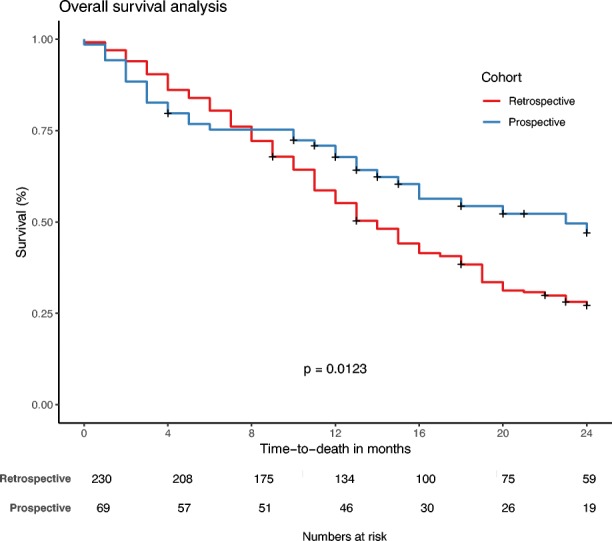
Kaplan–Meier analysis of the difference in overall survival between the retrospective and the prospective cohorts. A survival benefit was observed in the prospective patient cohort (*p* = 0.012)

In the retrospective cohort, Kaplan–Meier analysis showed no significant differences in OS or PFS between patients who were and were not PET/CT-staged (*p* = 0.867 and 0.304, respectively; Fig. 1). Only 18.1% of the retrospective data were censored; in contrast, 52.2% of the prospective data were censored. Median survival was 14 months (95% CI 12–15 months) in the retrospective cohort and 23 months (95% CI 15–30 months) in the prospective cohort (*p* = 0.012, log-rank test). Two-year OS was 27% in the retrospective cohort and and 47% in the prospective cohort. The corresponding Kaplan–Meier analysis is shown in Fig. 2.

**Fig. 3:**
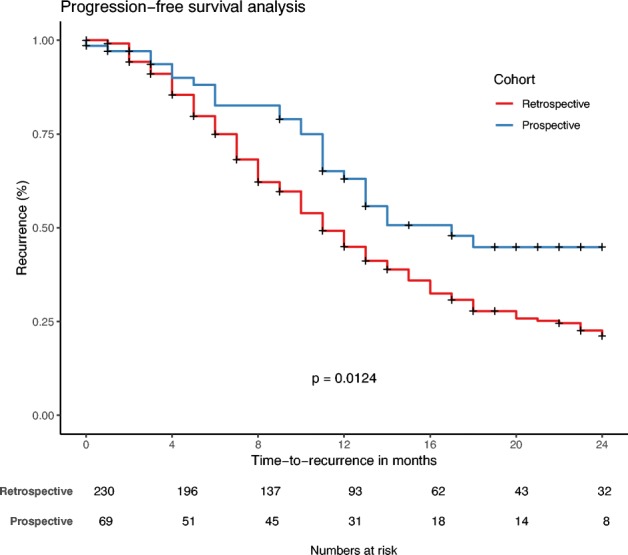
Kaplan–Meier analysis of the difference in progression-free survival between the retrospective and the prospective cohort. A survival benefit was observed in the prospective patient cohort (*p* = 0.012)

Kaplan–Meier analysis of PFS in the retrospective and the prospective cohorts is shown in Fig. 3. Median time to progression was 11 months (95% CI 9–12 months) in the retrospective cohort and 17 months (95% CI 10–23 months) in the prospective cohort (*p* = 0.012, log-rank test). Two-year PFS was 22% in the retrospective cohort and 45% in the prospective cohort.

## Discussion

This study investigated the impact of introducing FDG PET/CT-guided concurrent CRT, supported by training and QC, on the OS in patients with stage III NSCLC. Preliminary results demonstrated a positive trend in a cohort comparison towards improved OS and PFS in the prospective cohort, suggesting a benefit from implementing FDG PET/CT-guided concurrent CRT in patients with stage III NSCLC in centres in LMIC with limited experience with PET/CT. TVD QC showed that IAEA study guidelines were implemented successfully in the clinic in 74% of patients. This demonstrates compliance with the study guidelines in the clinic, but also emphasizes the importance of QC in multi-centre trials to ensure compliance with the study protocol. Using TVD QC we were therefore able to confirm compliance with IAEA study guidelines in the clinic. This procedure led to the removal of five patients ineligible for curative CCRT who could otherwise have influenced the outcome of this study. Four issues were observed in TVD QC: incorrect staging, involved lymph nodes not included, tumours too large to treat radically without exceeding dose constraints, and tumours not delineated following the reGTV approach. The QC reviews continued to inform study participants during patient accrual and served as educational material when these issues occurred, which emphasizes once more the importance of QC during clinical studies.

Significant differences in TNM stage were observed between the retrospective and prospective cohorts. The retrospective cohort included predominantly stage IIIA patients, whereas the prospective cohort had a more balanced distribution of patients with stages IIIA, B and C. Despite a survival benefit of stage IIIA over IIIB and IIIC, results demonstrated better survival in the prospective cohort. Nevertheless, 48% of the patients in the retrospective cohort were not PET/CT-staged, and could have been incorrectly staged. Indeed, in all centres, a high percentage (up to 51%) of patients were upstaged to stage IV after staging with PET/CT became the standard.

The improved OS and PFS were possibly due to several factors. Besides the introduction of PET/CT for TVD and improved patient selection with FDG PET/CT, patients in the prospective cohort all received CCRT (64% in the retrospective cohort; 100% in the prospective cohort) and were also treated with more advanced RT techniques such as IMRT and VMAT (14% in the retrospective cohort; 45% in the prospective cohort), which could also have led to survival benefits [6]. Even so, there was no significant difference in the prescribed doses between the cohorts, and evidence is lacking on the survival benefit of IMRT/VMAT versus 3DCT in lung cancer patients [29]. On the other hand, more confidence was gained in PET/CT-based contouring, increasing delineation accuracy which hopefully resulted in reduced geographic miss of tumour. This may explain the improved local control seen in Fig. 2. In addition, the PERTAIN trial improved or reaffirmed collaborative working relationships between nuclear medicine and radiation oncology departments in the participating centres. This collaboration may not only have led to improved delineation, but also may have improved patient management by streamlining the patient pathway from diagnosis to treatment. Evaluation of the impact of this collaboration on outcome was beyond the scope of this study, and further research is required to obtain definitive evidence [30, 31].

In the PERTAIN trial there was a heterogeneous group of participating centres with different levels of experience in PET/CT scan acquisition. Furthermore, the training interventions were limited to RO and NMP chief scientific investigators. A train-the-trainers approach was used to disseminate the knowledge further in the departments involved in the PERTAIN study. The improvement in survival outcomes shown in this analysis suggests that this training approach had a clinically meaningful impact in the participating centres. We suggest that it is feasible to disseminate education regarding new radiation oncology techniques using the multiple intervention method we used [17].

One potential confounding impact in the comparison of outcomes between the cohorts may have been the impact of PET/CT staging alone. It is interesting to note that in the retrospective cohort, no significant difference in OS was observed between patients who were and were not PET/CT-staged (Fig. 1), and hence this confounding effect may have been negligible in this cohort, but it is acknowledged that because of the size of these groups, the study may not have been powered to detect a true difference. Another potential confounding factor was the selection bias that may have been present between the retrospective cohort and prospective cohort with regard to patients who died during treatment. This may explain the worse survival seen in the first months in the prospective cohort. Five patients died during RT in the prospective cohort after being included in the analysis, and patients who died during treatment were not selected for the retrospective cohort. However, this would have had the effect of reducing the apparent survival difference between the two arms of the study. Another influence that may have contributed to worse survival in the first months may have been the higher incidence of smokers and patients with COPD in the prospective cohort.

### Conclusion

The initial analysis of the PERTAIN study showed that a combined package of FDG PET/CT-planned RT, the routine use of concurrent CRT with training support, and a robust QC process led to improved OS and PFS in patients with stage III NSCLC patients in low- and middle-income countries.

## Electronic supplementary material

ESM 1 (DOCX 3904 kb)
