# Supplementary material for: Introducing FDG PET/CT-guided chemoradiotherapy for stage III NSCLC in low- and middle-income countries: preliminary results from the IAEA PERTAIN trial
Source: Eur J Nucl Med Mol Imaging. 2019 Jul 31;46(11):2235–43. doi: 10.1007/s00259-019-04421-5 (PMC6717604; doi:10.1007/s00259-019-04421-5)

# Supplementary Material S1.

In the retrospective study, a total of five different eCRFs were used to collect patient data. In Form 1, eligibility criteria of each patient were checks whereas Form 2 consisted of detailed information on delivered treatment. Form 3 was used in case the patient was alive and did not finish two-year follow up, and provided data on survival status. Otherwise, Form 4 was filled in and consisted of information on survival and cause of death. As for the prospective part, Form 2 was filled in before - and Form 3 after treatment, to check for possible discrepancies between prescribed and delivered treatment. Form 4 contained survival data on follow-up moments (3, 6, 9, 12, 24 months). Form 5 was filled in if the patient went off-study for any reason or if the patient completed 24 months of follow-up.

**Table 1.** Overview of the electronic case report forms used to collect patient data. In both cohorts, Form 1 was used to check eligibility criteria.

|  | Retrospective | Prospective |
| --- | --- | --- |
| Form 1 | Checking for eligibility: inclusion/exclusion criteria | Checking for eligibility: inclusion/exclusion criteria |
| Form 2 | Treatment data | Pre-treatment data |
| Form 3 | Follow up data | Treatment data |
| Form 4 | Off-study data | Follow-up data |
| Form 5 | n/a | Off study data |


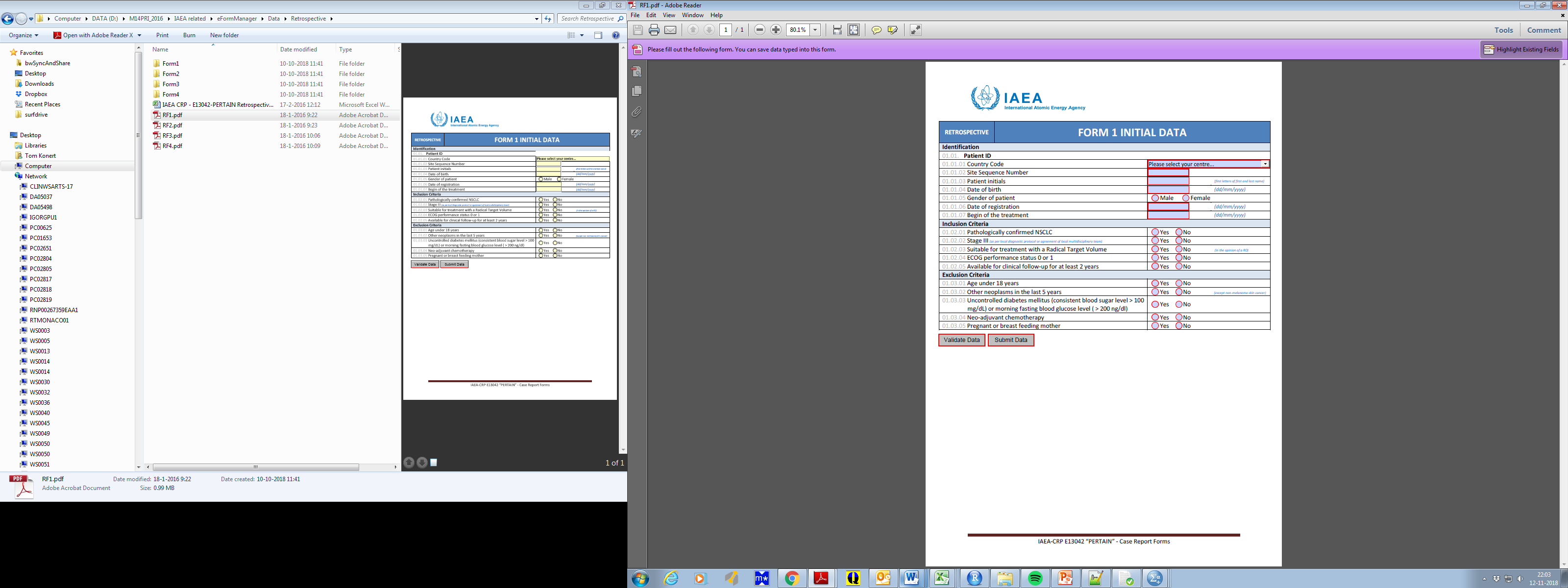


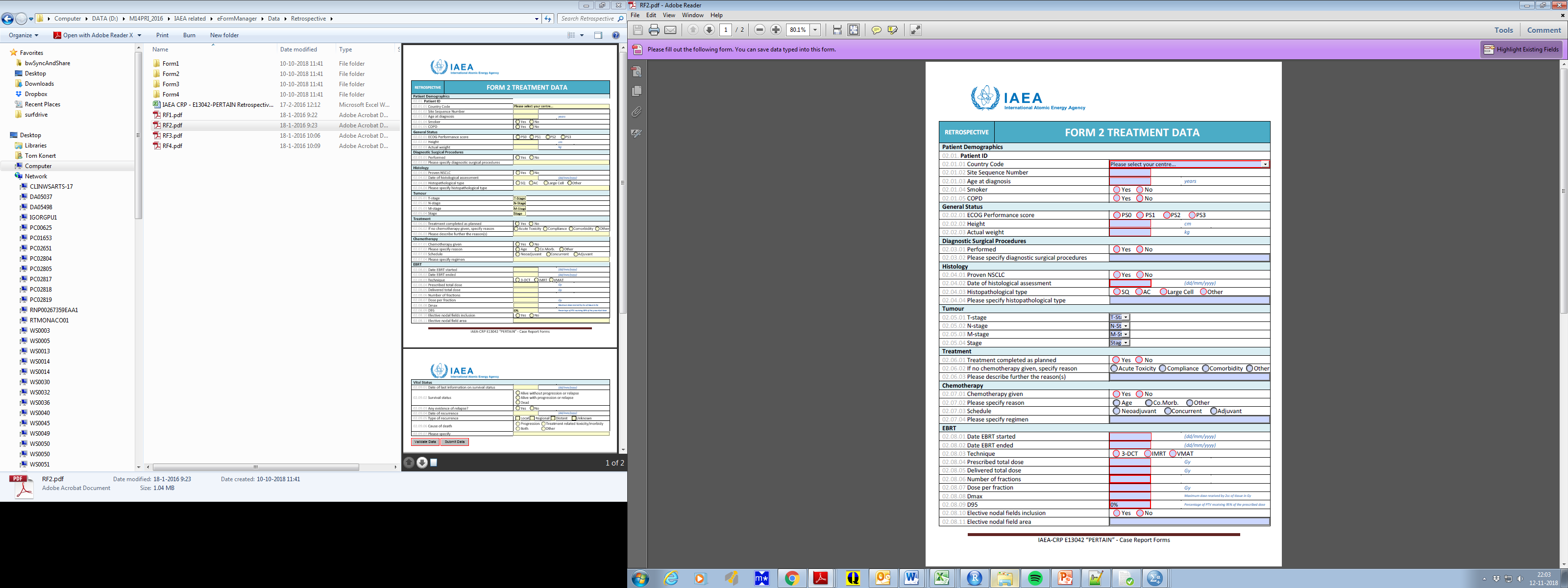


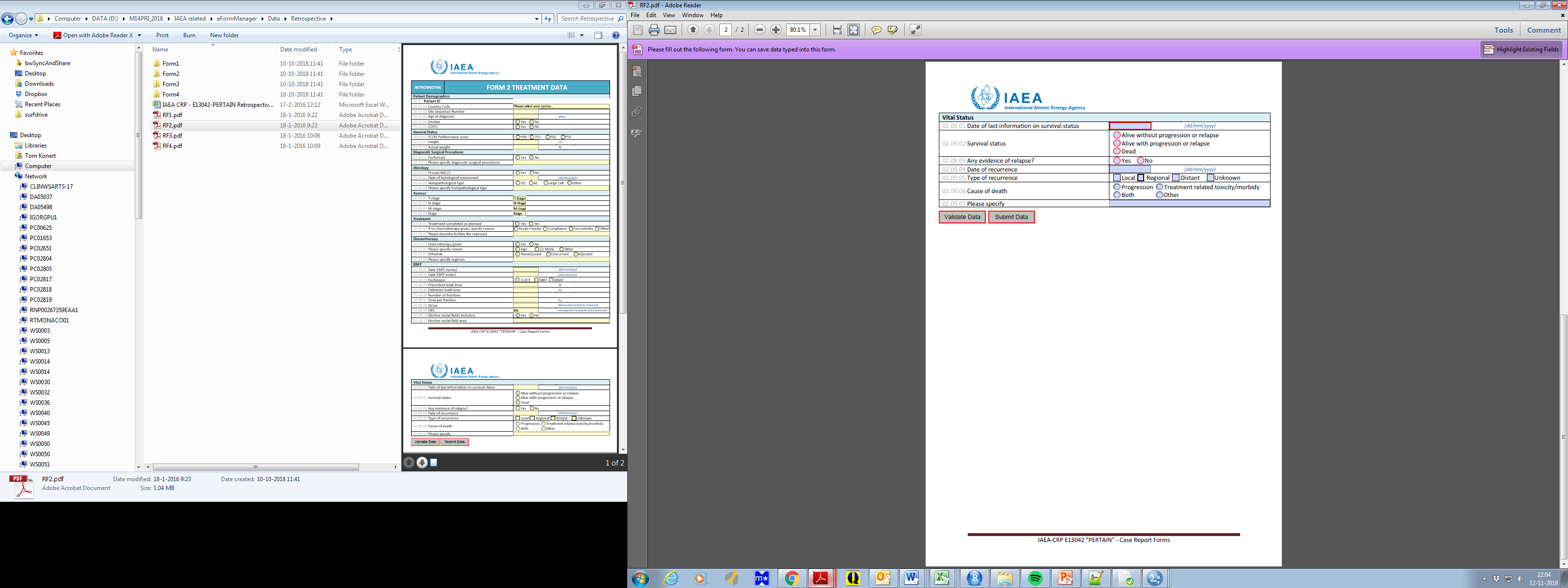


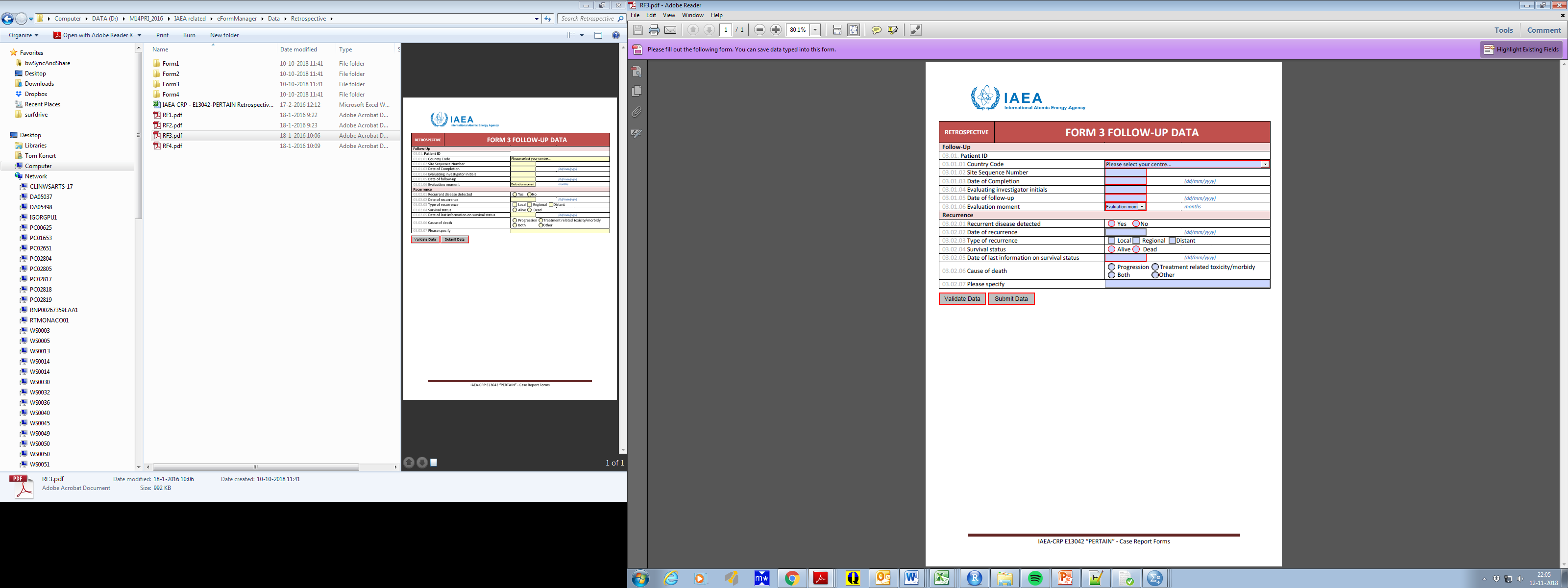


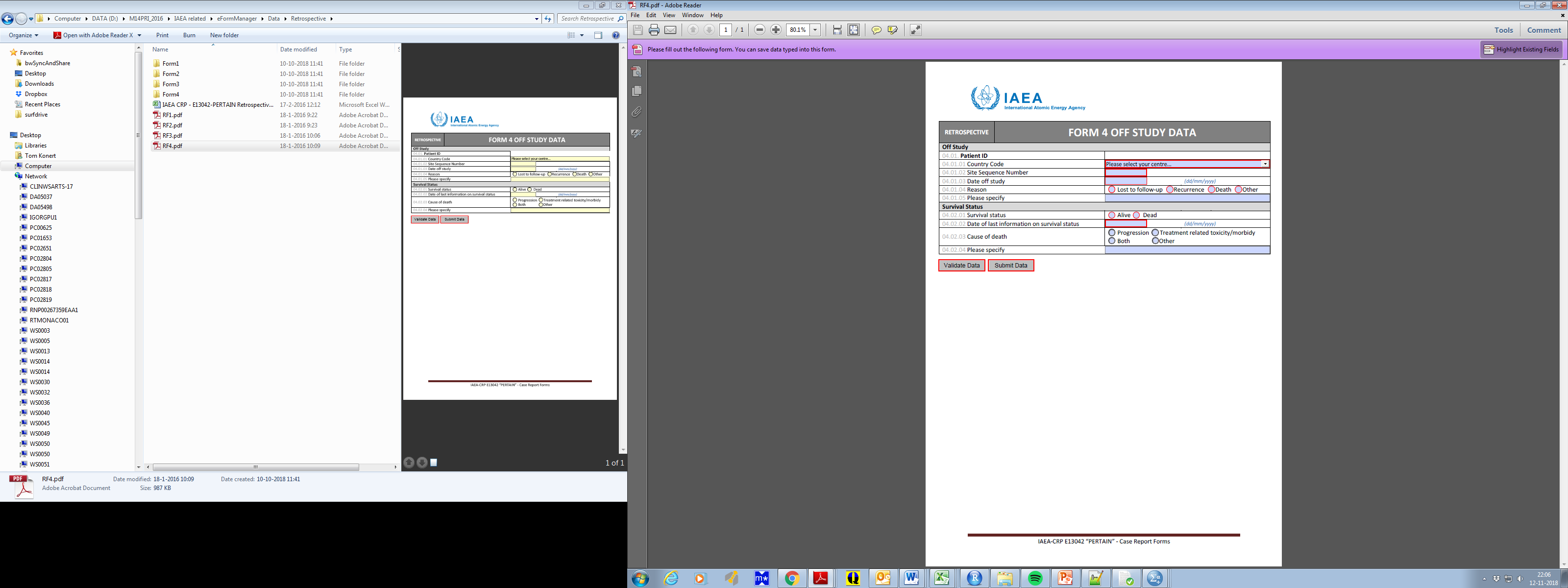


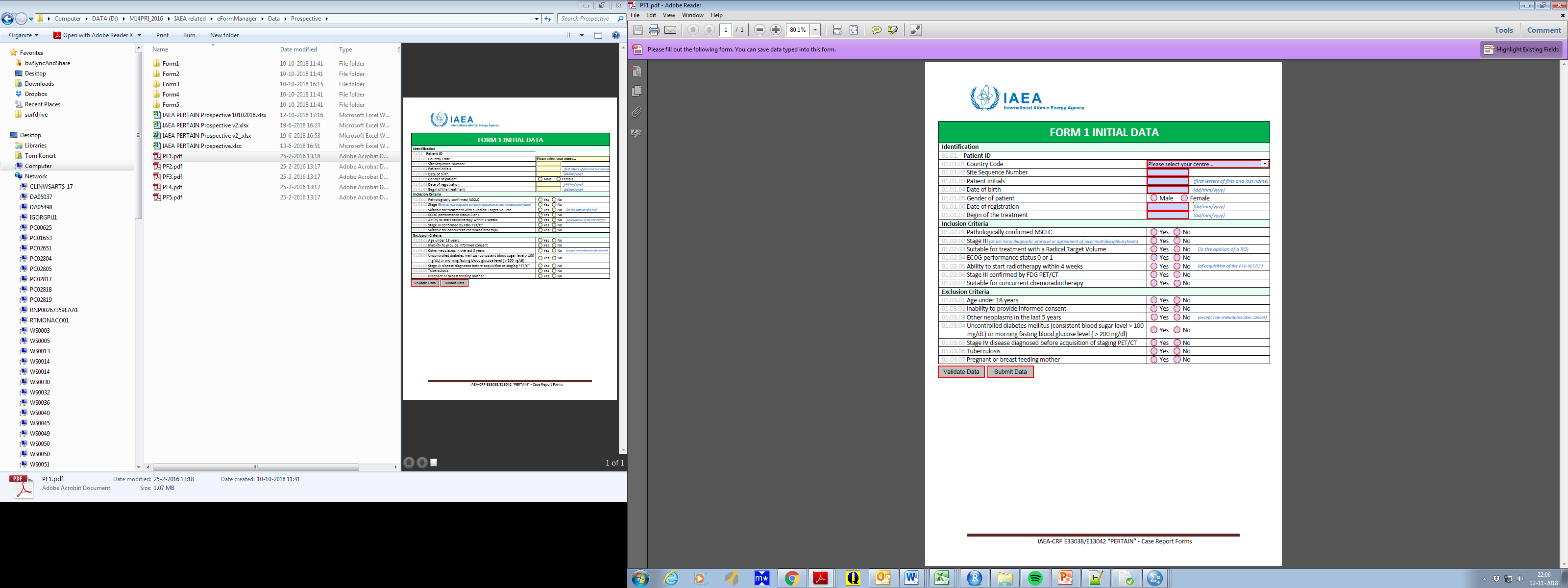


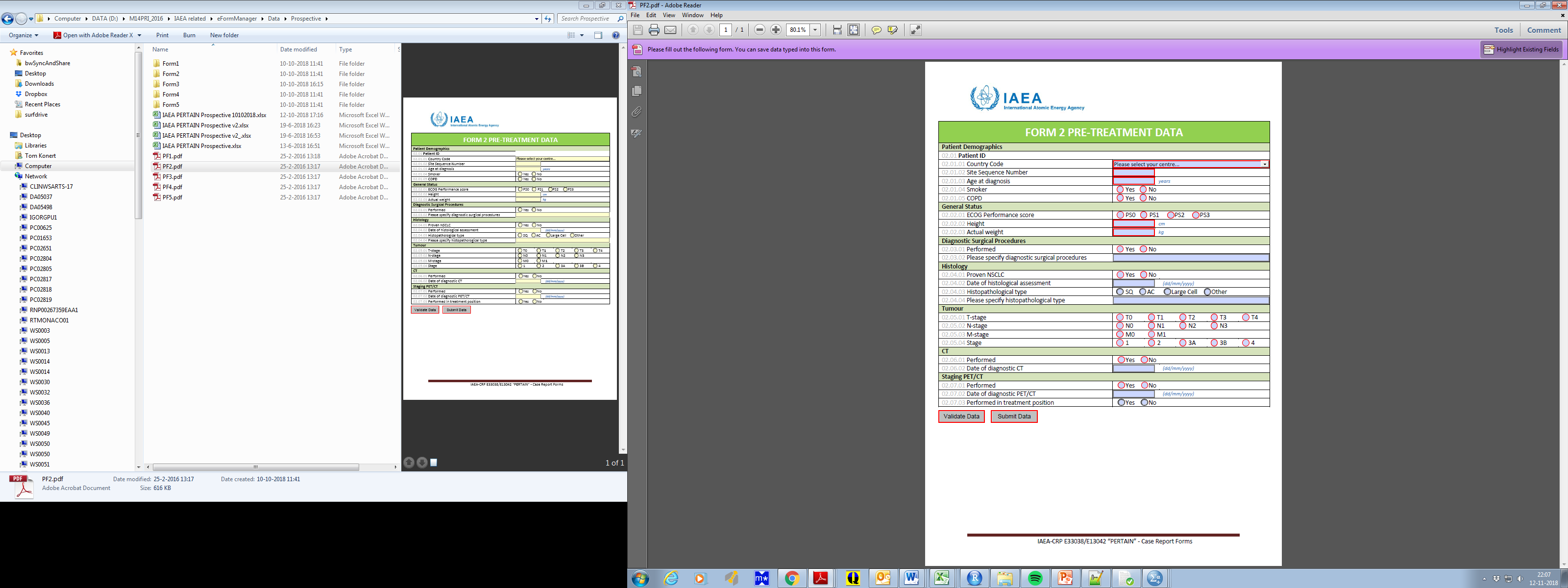


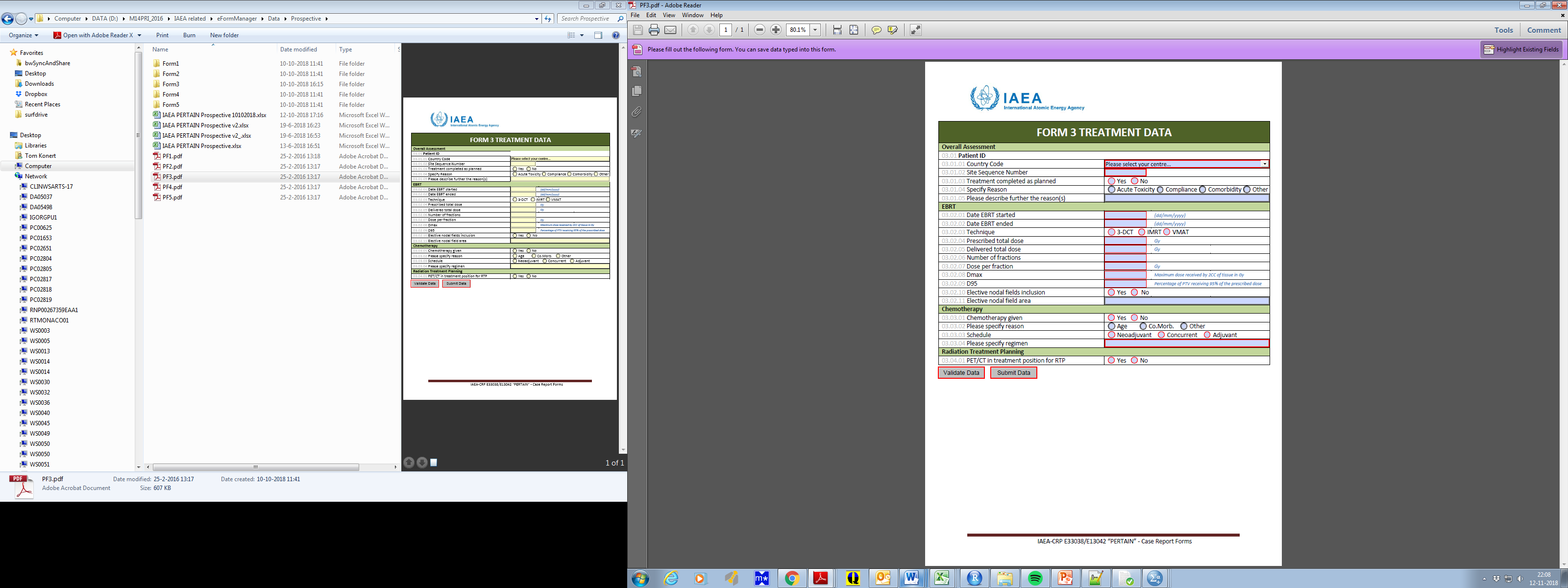


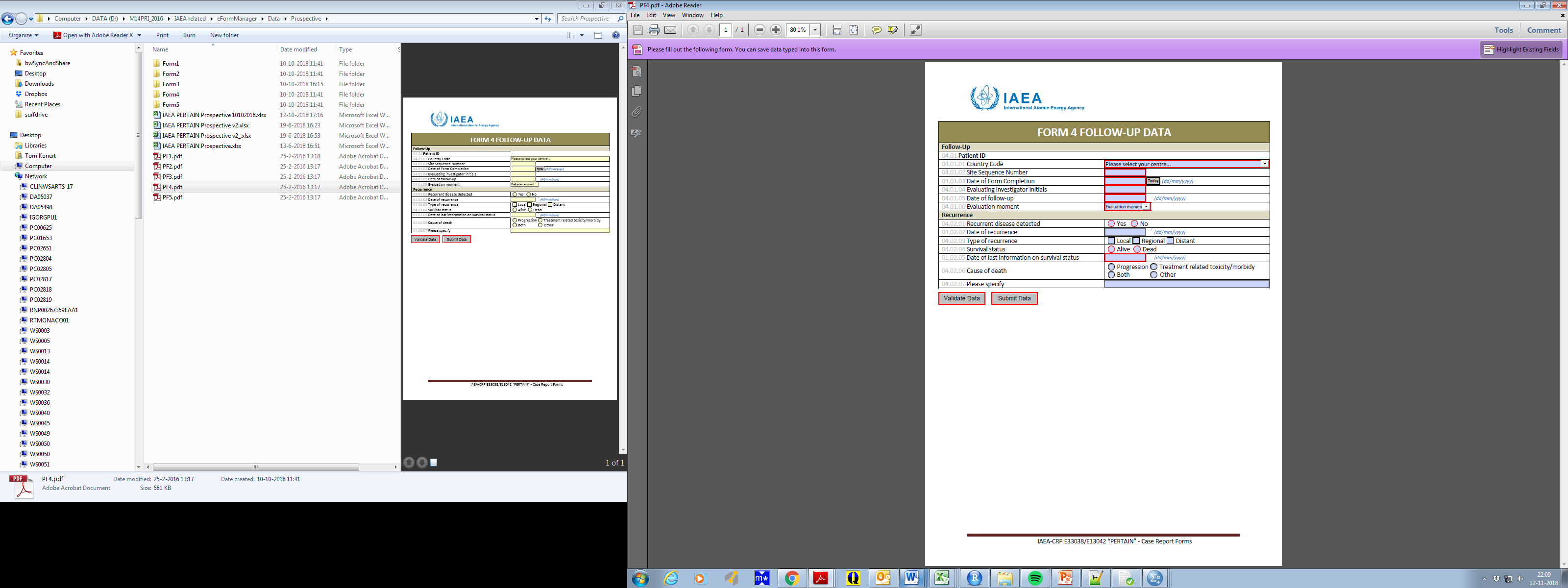


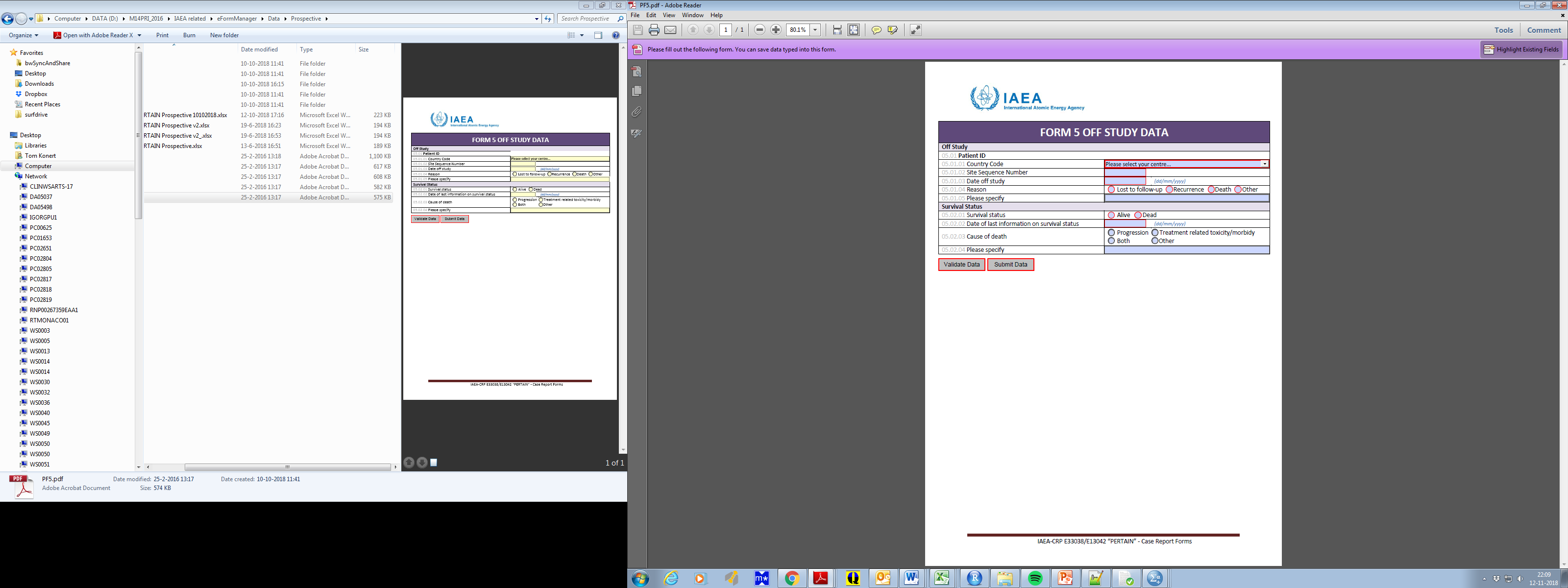

Supplement: Supplementary file 1 — (DOCX 3904 kb) [file 259_2019_4421_MOESM1_ESM.docx]
